# Dynamic profiling of intact glucosinolates in radish by combining UHPLC-HRMS/MS and UHPLC-QqQ-MS/MS

**DOI:** 10.3389/fpls.2023.1216682

**Published:** 2023-07-05

**Authors:** Chenghuan Yan, Yan Huang, Shuting Zhang, Lei Cui, Zhenbiao Jiao, Zhaoxin Peng, Xiaozhou Luo, Yun Liu, Zhengming Qiu

**Affiliations:** ^1^ Key Laboratory of Vegetable Ecological Cultivation on Highland, Ministry of Agriculture and Rural Affairs, Institute of Economic Crops, Hubei Academy of Agricultural Sciences, Wuhan, Hubei, China; ^2^ Hubei Key Laboratory of Vegetable Germplasm Enhancement and Genetic Improvement, Institute of Economic Crops, Hubei Academy of Agricultural Sciences, Wuhan, Hubei, China; ^3^ National Key Laboratory for Germplasm Innovation & Utilization of Horticultural Crops, Huazhong Agricultural University, Wuhan, Hubei, China; ^4^ Center for Synthetic Biochemistry, Shenzhen Institute of Synthetic Biology, Shenzhen Institutes of Advanced Technology, Chinese Academy of Sciences, Shenzhen, China

**Keywords:** radish, glucosinolates, functional foods, UHPLC-HRMS/MS, UHPLC-QqQ-MS/MS

## Abstract

Glucosinolates (GSLs) and their degradation products in radish confer plant defense, promote human health, and generate pungent flavor. However, the intact GSLs in radish have not been investigated comprehensively yet. Here, an accurate qualitative and quantitative analyses of 15 intact GSLs from radish, including four major GSLs of glucoraphasatin (GRH), glucoerucin (GER), glucoraphenin (GRE), and 4-methoxyglucobrassicin (4MGBS), were conducted using UHPLC-HRMS/MS in combination with UHPLC-QqQ-MS/MS. Simultaneously, three isomers of hexyl GSL, 3-methylpentyl GSL, and 4-methylpentyl GSL were identified in radish. The highest content of GSLs was up to 232.46 μmol/g DW at the 42 DAG stage in the ‘SQY’ taproot, with an approximately 184.49-fold increase compared to the lowest content in another sample. That the GSLs content in the taproots of two radishes fluctuated in a similar pattern throughout the five vegetative growth stages according to the metabolic profiling, whereas the GSLs content in the ‘55’ leaf steadily decreased over the same period. Additionally, the proposed biosynthetic pathways of radish-specific GSLs were elucidated in this study. Our findings will provide an abundance of qualitative and quantitative data on intact GSLs, as well as a method for detecting GSLs, thus providing direction for the scientific progress and practical utilization of GSLs in radish.

## 1 Introduction

Glucosinolates (GSLs), as sulfur- and nitrogen-containing secondary metabolites, are widely present in Brassicales plants, such as broccoli (*Brassica oleracea* var. *italica*), cauliflower (*B. oleracea* var. *botrytis*), and rocket salad (*Eurca sativa*) (Barco and Clay, 2019; Qin et al., 2023). Based on their side chains, GSLs can be classified into three types aliphatic, indolic, and aromatic GSLs (Halkier and Gershenzon, 2006). GSLs and their degradation products can protect plants from diseases and pests, promote the formation of flavor in cruciferous species, and provide anti-cancer and anti-inflammatory functions for human beings (Abdull Razis et al., 2011; de Oliveira et al., 2018). To date, more than 130 GSLs have been identified in plants (Petersen et al., 2018). The diversity of types and content abundance of GSLs in cruciferous plants contribute to their availability and the development of GSL-rich foods in the future.

Currently, the common GSL detection method is based on chromatography technology, which necessitates sulfatase desulfonation (Yi et al., 2016). Desulfonation reduces the polarity of GSLs, which is beneficial for GSL separation by reverse-phase chromatography and their detection by ultraviolet (UV) or diode-array detection (DAD). However, the procedures of removing the SO_3_^−^ group from intact GSLs are cumbersome and time-consuming. Moreover, the qualitative analysis of individual GSLs was affected adversely by desulfonation and low detection sensitivity of chromatography and spectroscopy (Yu et al., 2022). Generally, the intact GSLs rather than the desulfonated GSLs can represent the quality and nutritional value of vegetables. Liquid chromatography-tandem mass spectrometry (LC-MS/MS) is regarded as a more powerful tool for qualitative and quantitative analyses of intact GSLs than high-performance liquid chromatography (HPLC) (Bello et al., 2018; Li et al., 2021). Ultra-high-performance liquid chromatography-high-resolution tandem mass spectrometry (UHPLC-HRMS/MS) is a more effective qualitative analysis platform for secondary metabolites, exhibiting the advantages of high sensitivity, high resolution, and fast detection over other methods (Marshall and Hendrickson, 2008). Meanwhile, ultra-high performance liquid chromatography coupled to triple quadrupole mass spectrometry (UHPLC-QqQ-MS/MS) is considered as a high-throughput analysis platform for metabolites analysis, with an advantage for quantitative detection (Collado-Gonzalez et al., 2015). Therefore, UHPLC-HRMS/MS combined with UHPLC-QqQ-MS/MS can provide more information to uncover the metabolic features of plant components, especially those for the intact GSLs in radish.

Radish (*Raphanus sativus*), also known as ‘*Laifu*’ in ancient China, is a popular taproot vegetable of cruciferous species worldwide. One of the most significant characteristics of radish is its pungent flavor. The degradation product of glucoraphasatin (4-methylthio-3-butenyl GSL, GRH) is 4-methylthio-3-butenyl isothiocyanates (4MTB-ITC), which is regarded as the main pungent compound in uncooked radish (Ishida et al., 2015). The radish-specific GRH is the predominant GSL component in radishes, accounting for more than 84.5% of the total GSLs (Yi et al., 2016). A total of 17 GSL components have been discovered from radish using the desulfonation method so far (Wang et al., 2022). Moreover, GRH in radish, as the primary GSL component, was considered to be health-promoting (Montaut et al., 2010). The existing three important studies of GSLs are restricted to the HPLC method to detect the desulfur-GSL structures in radish (Ishida et al., 2015; Yi et al., 2016; Wang et al., 2022). Therefore, a simple and accurate approach for intact GSL detection is urgently required for screening radish germplasm and developing functional foods.

In this study, an integrated LC-MS/MS method, including UHPLC-HRMS/MS and UHPLC-QqQ-MS/MS, was employed for qualitative and quantitative analyses of intact GSLs in radish. Using the LC-MS/MS method, the metabolic characteristics of total GSLs from taproot and leaf tissues of two radish accessions ‘SQY’ and ‘55’ were investigated during five developmental stages. The fulfillment of this study will provide a reliable approach for the high-throughput detection of intact GSLs, and it will lay a foundation for the subsequent investigation of GSL variants in different genotypes, tissues, and developmental stages in radishes.

## 2 Materials and methods

### 2.1 Plant materials

**Figure 1:**
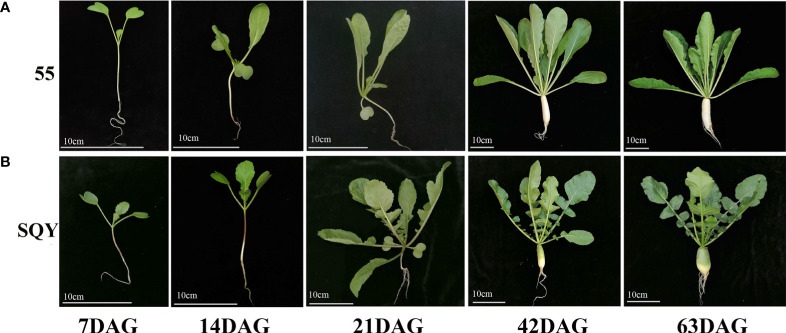
Five vegetative growth stages of two radish accessions ‘55’ **(A)** and ‘SQY’ **(B)**. DAG, day after germination. Bar scale = 10 cm.

Two Asian big radish accessions (*R. sativus* var. *hortensis*), ‘55’ (**Figure 1A**) and ‘SQY’ (**Figure 1B**), were used to identify and quantify GSLs. The ‘55’ was a typical white radish, and the ‘SQY’ was a radish with a partial green cortex and flesh. In autumn 2020, all the samples were cultivated in a plastic greenhouse of the base ‘Yangjiayan’ of Hubei Academy of Agricultural Sciences (Wuhan, China). Two radish tissues (leaf and taproot) during five different vegetative growth stages, including 7 DAG (day after germination), 14 DAG, 21 DAG, 42 DAG, and 63 DAG, were independently sampled (~ 10 g) and immersed in liquid nitrogen immediately, respectively. The samples were stored at –80°C freezer. The experiments were performed with three biological replicates for each sample.

### 2.2 Pretreatment of samples for GSLs detection

The collected samples were freeze-dried, ground into powder, and stored at -80°C freezer for further assay. According to the previously reported method, the GSLs were extracted with minor modifications (Li et al., 2021). The lyophilized powder samples (0.1g) were sequentially added with 1 mL HPLC-grade methanol (Thermo Fisher Scientific, Massachusetts, USA) solution (80%, v/v) preheated to 70 °C, and 5 μL 20mM internal standard sinigrin (Tokyo Chemical Industry Co., Tokyo, Japan). Then, these samples were vortexed for 1 min, and myrosinase activity was inhibited in a water bath at 70°C for 15 min. Next, the mixed samples were used for extraction on a shaker at 200 rpm for 15 min. After 15 min centrifugation at 13000 rpm, the supernatant was collected as much as possible into a 1.5 mL centrifuge tube. After the repeated centrifugation for 5 min, 500 μL supernatant was collected and transferred into a new 1.5 mL centrifuge tube. The 40 μL supernatant for each radish sample was mixed to act as QC (quality control) sample. The QC sample and residual supernatant were evaporated by a vacuum concentrator (Eppendorf, Hamburg, Germany), and dissolved again in 200 μL methanol (10%, v/v). Finally, the sample solution was filtered by a 0.22 μm syringe filter (SHIMADZU, Kyoto, Japan) and stored at -80°C freezer for further detection.

### 2.3 Qualitative analysis of GSLs by UHPLC-HRMS/MS

Qualitative analysis was performed on a Thermo Fisher Q Exactive (QE) Plus mass spectrometer (Thermo Fisher Scientific, Massachusetts, USA) equipped with a Thermo Scientific™ UltiMate™ 3000 UHPLC system. The Shim-pack GIST C18 column (2.1 mm × 100 mm, 1.9 µm; SHIMADZU, Kyoto, Japan) was used to separate different GSL components. A phase was ddH_2_O (0.1% formic acid, v/v), and B phase was 90% methanol (0.1% formic acid, v/v). The gradients of mobile phases were as follows: 0.00 min, 99% A and 1% B; 1.00 min, 99% A and 1% B; 8.00 min, 50% A and 50% B; 11.00 min, 10% A and 90% B; 12.00 min, 10% A and 90% B; 12.10 min, 99% A and 1% B; 14.00 min, 99% A and 1% B. The flow rate was 0.3 mL/min in the separation process with a 2 μL injection volume. The column temperature was maintained at 40°C. The GSLs were detected by the mass spectrometer using an electrospray ionization source (ESI) in a negative ion mode with *m/z* ranging from 100 to 1050. Other key parameters of the mass spectrometer were as follows: spray voltage, 3.0 kV; sheath gas flow, 40 bar; capillary temperature, 350°C; Aux gas heater temperature, 350°C; and S-lens RF level, 55 eV.

To establish the database of GSL components in radish, we prepared six QC samples including an equal amount of leaf samples and taproot samples for qualitative analysis. The mass spectrum data were collected by Xcalibur 3.0 software (Thermo Fisher Scientific, Massachusetts, USA) for subsequent analysis. The intact GSLs were identified and annotated using Compound Discoverer 4.0 software (Thermo Fisher Scientific, Massachusetts, USA) according to the *m/z* of molecular ions, ion fragments, and retention time (RT). Accurate mass data required that the error value should be lower than 5 ppm.

### 2.4 Quantitative analysis of GSLs by UHPLC-QqQ-MS/MS

The quantitative analysis of GSLs was performed using the Thermo Fisher TSQ Altis (Thermo Fisher Scientific, Massachusetts, USA). The UHPLC analysis was conducted by the same method as the above-mentioned qualitative analysis. The QqQ-MS parameters were as follows: spray voltage, 4.0 kV (+) and 3.5 kV (-); sheath gas flow, 40 bar; Aux gas flow, 10 bar; Ion transfer tube temperature, 350°C; and collision energy, 23 eV. The mass spectrum data were collected by Xcalibur 3.0 software, and further quantitative analysis was performed using Tracefinder 4.0 (Thermo Fisher Scientific, Massachusetts, USA). The GSL content was calculated according to the following formula: Amount _[target GSLs]_ (µmol g^-1^ DW) = Area _[target GSLs]_/Area _[internal standard]_ × Amount _[internal standard]_ (Mi et al., 2018). To control the quality of detection, two QC samples and a blank sample were detected after the determination of every 12 samples.

### 2.5 Statistical analysis

In this study, the data were processed by MetaboAnalyst 5.0 (Pang et al., 2021), and expressed by mean ± standard deviation (SD). The graphs were drawn by GraphPad Prism 8.0 software (GraphPad Software Inc., California, USA). GSL content was visualized by a heatmap plotted by TBtools software (Chen et al., 2020).

## 3 Results

### 3.1 Establishment of an integrated method for the determination of intact GSLs in radish

**Figure 2:**
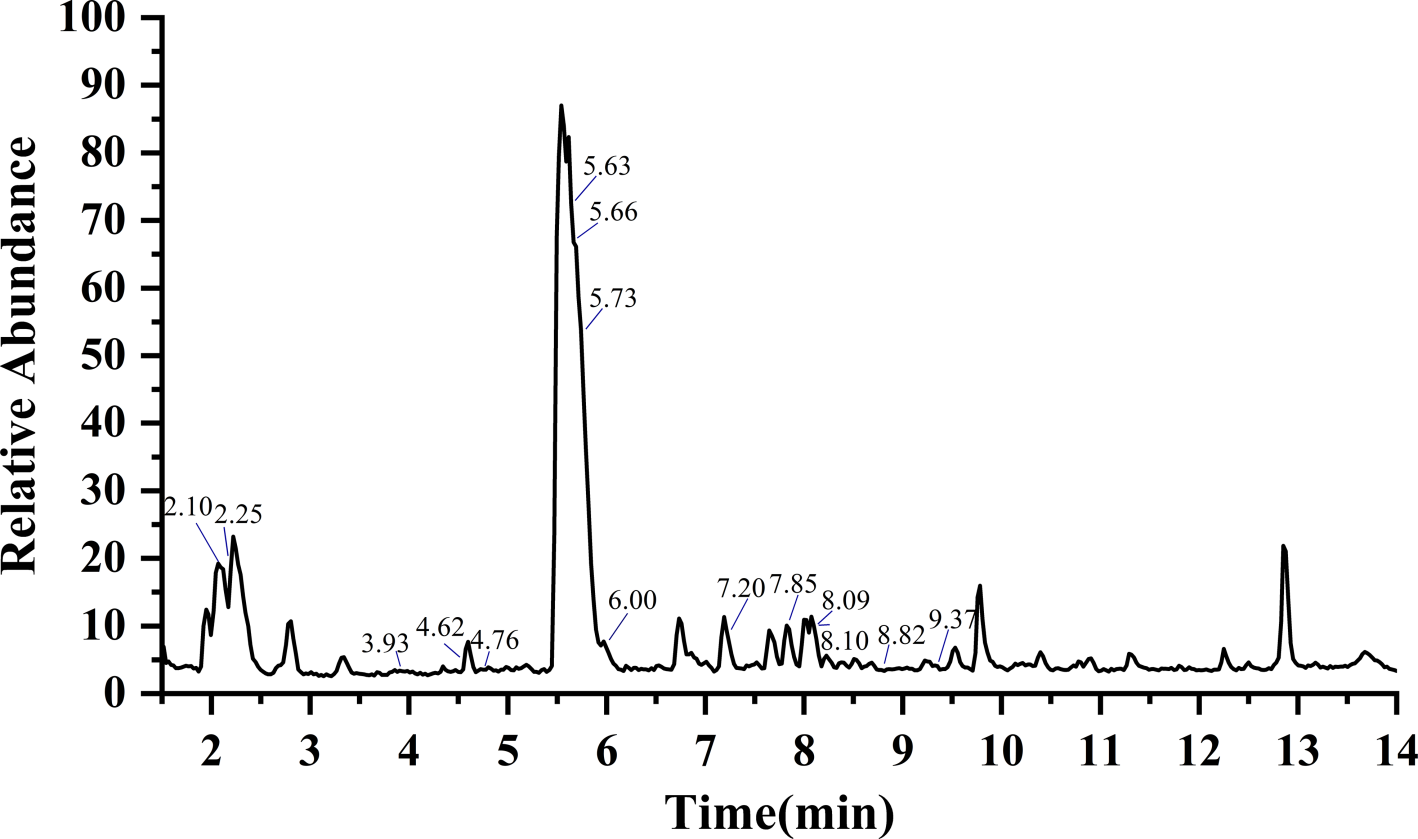
Total ion chromatogram (TIC) of GSLs in radish by UHPLC-HRMS/MS. According to the retention time, the marked GSLs is glucoraphanin, glucoraphenin, glucoalyssin, glucoiberverin, glucoerucin, glucoraphasatin, 3-methylpentyl GSL, 4-methylpentyl GSL, hexyl GSL, heptyl GSL, 1-hydroxyglucobrassicin, 4-hydroxyglucobrassicin, glucobrassicin, 4-methoxyglucobrassicin and neoglucobrassicin in turn. Details of individual GSLs were exhibited in **Table 1**.

To analyze the intact GSLs in radish, the LC-MS/MS method was established. In the pretreatment process, we used 10% methanol solution to dissolve the GSL extract for further UHPLC assay. Mobile phase H_2_O (A) and 90% methanol (B) were found to be a better combination than H_2_O (A) and acetonitrile (B) since the former showed a weaker solvent effect. In the negative ion mode and higher energy collision-induced dissociation (HCD) mode, the fragment ions of GSL components have a strong response. Thus, the intact GSLs in radish were apparently separated and accurately identified by UHPLC-HRMS/MS under a suitable chromatographic condition (**Figure 2**). Since different GSLs have a common basic structure containing a *D*-thioglucose group linked to a sulfonated aldoxime group and a variable side chain (-R) derived from amino acids. Different GSLs could produce some same fragment ions such as *m/z* 274.9908 (Glu-S-SO_3_^-^), *m/z* 259.0139 (Glu-SO_4_^-^), *m/z* 195.0333 (Glu-S^-^), *m/z* 119.0345 (C_4_H_7_O_4_^-^), *m/z* 96.9588 (HSO_4_^-^), *m/z* 95.9511 (SO_4_^2-^), *m/z* 79.9561 (SO_3_^2-^), and *m/z* 74.9897(C_2_H_3_SO^-^). As the internal standard, sinigrin had one molecular ion ([M-H]^-^, *m/z* 358.0277) and the above-mentioned eight fragment ions (**Figure S1**). Based on the fragmentation patterns, *m/z* 119.0345 (C_4_H_7_O_4_^-^) and *m/z* 74.9897 (C_2_H_3_SO^-^) have been produced by the cleavage of *m/z* 195.0333 (Glu-S^-^), while *m/z* 96.9601 (HSO_4_^-^), *m/z* 95.9523 (SO_4_^2-^), and *m/z* 79.9562 (SO_3_^2-^) were formed by the cleavage of the sulfonated aldoxime group (Bialecki et al., 2010). In terms of fragmentation patterns and response abundance, we selected *m/z* 259.0139, *m/z* 96.9588, *m/z* 95.9511, and *m/z* 74.9897 as characteristic product ions to further identify GSL components. Furthermore, UHPLC-QqQ-MS/MS analysis was performed to accurately quantify GSL components with the corresponding parameters set presented in **Table S1**. We optimized several selected reaction monitoring (SRM) transitions to analyze each GSL component. Then, each precursor ion and the corresponding selected fragment ion (m/z 96.959, the most intense transition) of GSLs were subjected to identification and quantification, respectively. Additionally, we calculated the total detection time to evaluate the efficiency of our method in this study (**Table S2**). Taken together, we established an integrated LC-MS/MS method for the identification and quantification of intact GSLs in radish.

### 3.2 Structures and mass fragments of individual GSL in radish

In this study, we identified the intact GSLs rather than desulfur-GSLs from radish using the UHPLC-HRMS/MS method. Firstly, we searched the database for molecular ions ([M-H]^-^) to obtain candidate GSLs by Compound Discoverer 4.0. Then, GSL components were identified based on the accurate *m/z* of molecular ion ([M-H]^-^) and four characteristic fragment ions of *m/z* 259.0139, *m/z* 96.9589, *m/z* 95.9511, and *m/z* 74.9897 as well as the specific fragment derived from the side chain of GSLs. Finally, a total of 15 GSL components were identified from six quality control (QC) samples of radish (**Figure 2**), of which 10 belonged to aliphatic GSLs and 5 were classified into indolic GSLs. The detailed information of these 15 GSL identified from radish was presented in **Table 1**, including types, name, molecular formula, retention time, *m/z*, and MS/MS fragments. The most abundant aliphatic type and indolic type of GSLs in radish were glucoraphasatin (GRH) and 4-methoxyglucobrassicin (4MGBS), respectively. As expected, aliphatic GRH ([M-H]^-^, *m/z* 418.0303) at 5.73 min was the prominent GSL in the QC samples. Although the retention time of glucoerucin (GER, 5.66 min), 4-hydroxyglucobrassicin (4HGBS, 5.63 min), and GRH (5.73 min) were very close, their different secondary modification, fragmentation patterns, and relative contents provided enough information to distinguish these three GSLs. The 4MGBS ([M-H]^-^, *m/z* 477.0641) was the most abundant indolic GSL that emerged at 7.20 min.

**Table 1:** The identified GSLs and detected mass fragments of two radish accessions.

Types	Chemical name	Common name and its abbreviation	Molecular formula	Retention time (min)	[M-H]^-^ (*m/z*)	MS/MS fragments
Aliphatic	4-methylsulfinylbutyl GSL	Glucoraphanin, GRA	C_12_H_23_NO_10_S_3_	2.10	436.0412	74.9897, 79.9501, 95.9511, 96.9589, 119.0430, 195.0326, 259.0130, 274.9622, 372.0434
Aliphatic	4-methylsulfinyl-3-butenyl GSL	Glucoraphenin, GRE	C_12_H_21_NO_10_S_3_	2.25	434.0252	74.9897, 79.9561, 95.9511, 96.9589, 119.0337, 195.0325, 259.0130, 274.9912, 354.0694, 370.0296
Aliphatic	5-methylsulfinylpentyl GSL	Glucoalyssin, GAL	C_13_H_25_NO_10_S_3_	3.93	450.0573	74.9897, 79.9561, 95.9511, 96.9589, 119.0343, 195.0339, 208.0474, 259.0130, 274.9899, 386.0592
Aliphatic	3-methylthiopropyl GSL	Glucoiberverin, GIV	C_11_H_21_NO_9_S_3_	4.76	406.0306	74.9897, 79.9561, 95.9511, 96.9589, 119.0345, 164.0203, 195.0333, 212.9711, 227.0239, 259.0130, 274.8939
Aliphatic	4-methylthiobutyl GSL	Glucoerucin, GER	C_12_H_23_NO_9_S_3_	5.66	420.0459	74.9897, 79.9561, 95.9511, 96.9589, 119.0342, 178.0157, 195.0322, 226.9890, 259.0132, 274.9918
Aliphatic	4-methylthio-3-butenyl GSL	Glucoraphasatin, GRH	C_12_H_21_NO_9_S_3_	5.73	418.0303	74.9897, 79.9561, 95.9511, 96.9589, 119.0339, 195.0321, 241.0032, 259.0140, 274.9895, 338.0718
Aliphatic	3-methylpentyl GSL	–	C_13_H_25_NO_9_S_2_	7.85	402.0897	74.9897, 79.9561, 95.9511, 96.9589, 195.0329, 259.0130, 274.9907
Aliphatic	4-methylpentyl GSL	–	C_13_H_25_NO_9_S_2_	8.09	402.0896	74.9897, 79.9561, 95.9511, 96.9589, 119.0337, 195.0329, 259.0130, 274.9906
Aliphatic	Hexyl GSL	–	C_13_H_25_NO_9_S_2_	8.82	402.0861	74.9897, 79.9559, 95.9511, 96.9601, 274.8944
Aliphatic	Heptyl GSL	–	C_14_H_27_NO_9_S_2_	9.37	416.1058	74.9897, 79.9561, 95.9511, 96.9589, 119.0340, 195.0332, 259.0130, 274.9894
Indolic	1-hydroxy-3-indolylmethyl GSL	1-hydroxyglucobrassicin, 1HGBS	C_16_H_20_N_2_O_10_S_2_	4.62	463.0485	74.9897, 79.9561, 95.9511, 96.9589, 119.0340, 195.0326, 259.0131, 274.9914
Indolic	4-hydroxy-3-indolylmethyl GSL	4-hydroxyglucobrassicin, 4HGBS	C_16_H_20_N_2_O_10_S_2_	5.63	463.0488	74.9898, 79.9560, 95.9511, 96.9589, 195.0312, 259.0138
Indolic	3-indolylmethyl GSL	Glucobrassicin, GBS	C_16_H_20_N_2_O_9_S_2_	6.00	447.0537	74.9897, 79.9561, 95.9511, 96.9589, 119.0342, 195.0330, 259.0140, 274.9905
Indolic	4-methoxy-3-indolylmethyl GSL	4-methoxyglucobrassicin, 4MGBS	C_17_H_22_N_2_O_10_S_2_	7.20	477.0641	74.9898, 79.9561, 95.9511, 96.9588, 119.0340, 195.0324, 259.0130, 274.9903
Indolic	1-methoxy-3-indolylmethyl GSL	Neoglucobrassicin, NEO	C_17_H_22_N_2_O_10_S_2_	8.10	477.0641	74.9898, 79.9561, 95.9511, 96.9589, 446.0462

**Figure 3:**
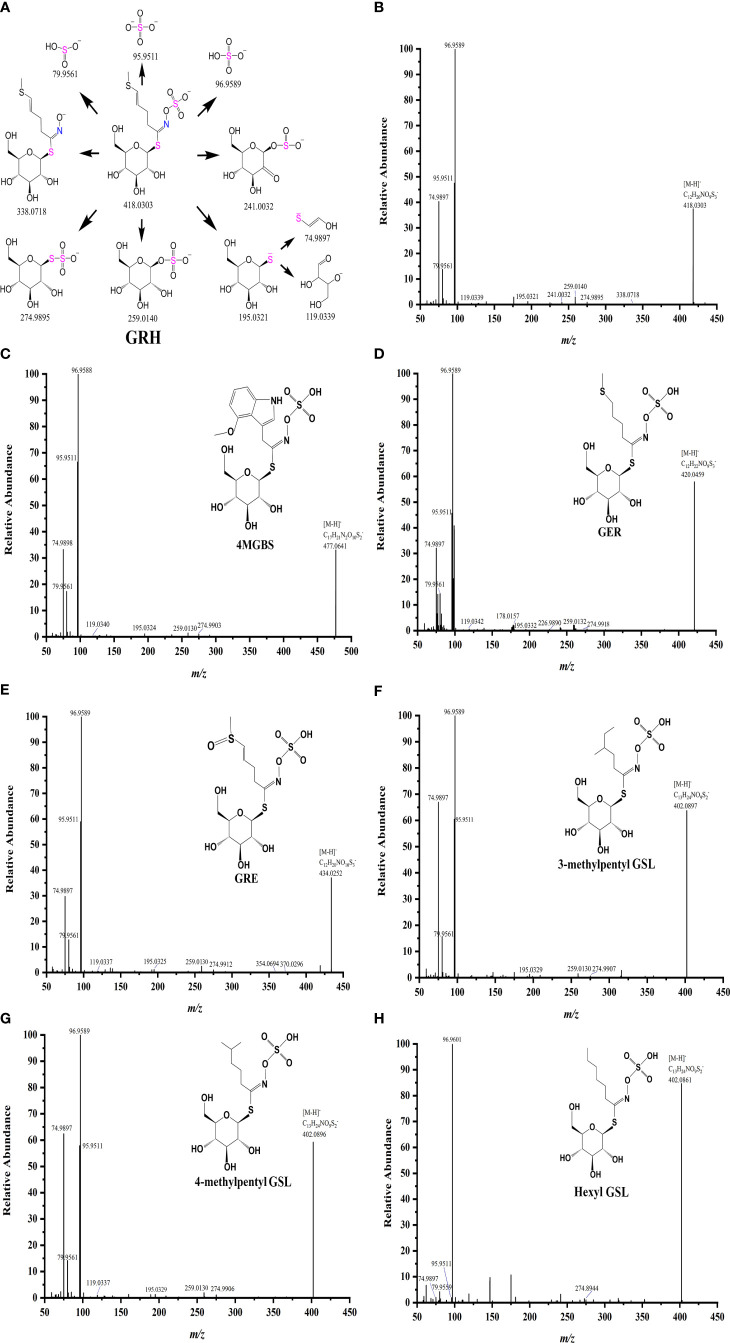
Fragmentation patterns of GRH **(A)**, and MS/MS spectrum of four major GSLs **(B–E)** and three isomers **(F–H)** in radish.

Further, we analyzed the MS/MS spectrum and identified characteristic fragment ions of side chains of GSLs (**Figures 3**, **S2**). The side chain variations and modifications of GSLs resulted in the production of some characteristic fragment ions. For example, the side chain of GRH with an unsaturated double bond generated a characteristic fragment ion *m/z* 338.0718 (Glu-S-NO-C_6_H_9_S^-^) (**Figures 3A, B**), which was consistent with previous studies (Maldini et al., 2017). Likewise, the glucoraphenin (GRE), GRH downstream product, formed the characteristic fragment ion of *m/z* 370.0296 (**Figure 3E**). Due to the loss of the methyl sulphoxide, the three GSLs produced their characteristic fragment ions, *m/z* 372.0434 for GRA, *m/z* 386.0592 for GAL, and *m/z* 354.0694 for GRE, respectively (Francisco et al., 2009). MS/MS data showed a loss of *m/z* 178.0355 vs. *m/z* 164.0203 (*Δ*=14) and *m/z* 226.9890 vs. *m/z* 212.9711 (*Δ*=14), which might be attributed to the fact that GER has one more -CH_2_- (*m/z* 14.0156) than the glucoiberverin (GIV) in the side chain. For the isomers NEO and 4MGBS, NEO eluted later than 4MGBS, and the bonds of -OCH_3_ of these two isomers broke more easily from N-methoxy than from C-methoxy (Dong et al., 2021). Our data indicated that the loss of -OCH_3_ led to the generation of *m/z* 446.0462 in NEO fragments (**Figure S2H**). Since the substance with high polarity was eluted first on the reverse column, the isomers with different side chain structures could be identified based on their polarities (Lelario et al., 2012). Meanwhile, we observed that the *m/z* values of three molecular ions [M-H]^-^ were 402.0897, 402.0896, and 402.0861, exhibiting the same molecular formula of C_13_H_25_NO_9_S_2_ (**Figures 3F–H**). Thus, we identified 3-methylpentyl GSL (7.85 min), 4-methylpentyl GSL (8.09 min), and hexyl GSL (8.82 min) as isomers.

### 3.3 Content of GSLs in radish

**Figure 4:**
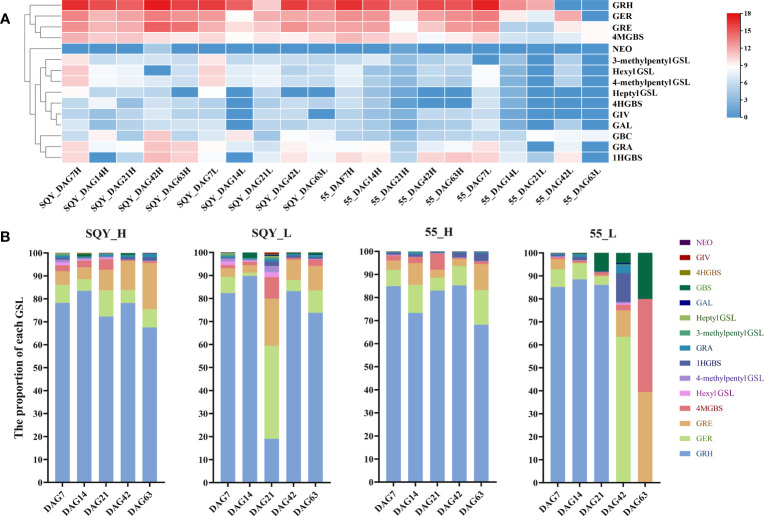
Heatmap **(A)** and percentage **(B)** of individual GSL components in two radish accessions ‘SQY’ and ‘55’. The GSL component contents were calculated by their log2-scaled relative abundance values. GRH, 4-methylthio-3-butenyl GSL; GRE, 4-methylsulfinyl-3-butenyl GSL; GER, 4-methylthiobutyl GSL; GRA, 4-methylsulfinylbutyl GSL; GIV, 3-methylthiopropyl GSL; GAL, 5-methylsulfinylpentyl GSL; GBS, 3-indolylmethyl GSL; 4HGBS, 4-hydroxy-3-indolylmethyl GSL; 1HGBS, 1-hydroxy-3-indolylmethyl GSL; 4MGBS, 4-methoxy-3-indolylmethyl GSL; NEO, 1-methoxy-3-indolylmethyl GSL. ‘H’, taproot tissue; ‘L’, leaf tissue; ‘DAG’, the day after germination; the numbers behind DAG represent the sampling date.

To clarify the fluctuation trend of GSL content, we conducted the quantitative analysis of intact GSLs during five vegetative growth periods from two radish accessions ‘SQY’ and ‘55’ using UHPLC-QqQ-MS/MS. The total content of GSLs in leaves ranged from 1.259 to 179.336 μmol g^-1^ dry weight (DW) during five developmental stages of two accessions, and the maximum value of total GSL content was approximately 142.44 folds of the minimum. A sharp variation in total GSL content was also observed in the taproots of ‘SQY’ and ‘55’, ranging from 11.990 to 232.458 μmol g^-1^ DW (**Table 2**). Heatmap showed that GRH, GER, GRE, and 4MGBS were the four high abundance (major) GSLs in radish (**Figure 4A**). In taproots, the content of GRH ranged from 9.955 to 181.903 μmol g^-1^ DW, GER from 0.678 to 13.182 μmol g^-1^ DW, GRE from 0.403 to 29.413 μmol g^-1^ DW, and 4MGBS from 0.317 to 3.410 μmol g^-1^ DW, accounting for approximately 67.55%-85.24%, 5.06%-15.03%, 3.03%-20.12%, and 0.44%-7.03% in the total GSLs, respectively (**Figure 4B**; **Table S3**). Unexpectedly, no GRH was detected in the leaves of ‘55’ at 42 DAG and 63 DAG, but the content of its downstream product GRE was 0.708 μmol g^-1^ DW at 42 DAG and 0.497μmol g^-1^ DW at 63 DAG, respectively. The low levels of GSLs in the leaves might be attributed to unknown transportation mechanisms in the ‘55’. Additionally, 4MGBS was the only indolic GSL derived from tryptophan (Trp) among the four major GSLs, and its concentration was relatively low in radish. The remaining 11 GSLs, such as 4HGBS, GRA, GAL, and GBS, were minor GSLs due to their low abundance. The content of these minor GSLs was nearly lower than 1 μmol g^-1^ DW in corresponding samples (**Table 2**). These results indicated that aliphatic GSLs were the predominant GSLs, and methionine (Met) was the main precursor of GSL biosynthesis in radish.

**Table 2:** Content of individual GSL (μmol g^-1^ DW) in two radish accessions during five vegetative growth stages.

samples	GRH	GER	GRE	Hexyl GSL	4-methylpentyl GSL	3-methylpentyl GSL	GAL	GIV	Heptyl GSL	GRA	4HGBS	NEO	1HGBS	4MGBS	GBS	Total GSLs
**55_DAG7H**	102.001 ± 25.926	8.409 ± 2.155	4.954 ± 1.371	0.182 ± 0.065	0.168 ± 0.065	0.056 ± 0.080	0.042 ± 0.032	0.031 ± 0.010	0.061 ± 0.028	0.347 ± 0.331	0.070 ± 0.009	ND	1.092 ± 0.165	2.687 ± 1.496	0.032 ± 0.010	120.134 ± 28.594
**55_DAG14H**	43.106 ± 28.393	7.206 ± 3.757	5.470 ± 2.649	0.013 ± 0.011	0.025 ± 0.018	0.125 ± 0.063	0.019 ± 0.013	0.012 ± 0.009	0.011 ± 0.009	0.483 ± 0.323	0.019 ± 0.012	ND	0.690 ± 0.426	1.583 ± 1.204	0.024 ± 0.021	58.786 ± 24.730
**55_DAG21H**	9.955 ± 6.199	0.678 ± 0.621	0.403 ± 0.220	0.008 ± 0.007	0.007 ± 0.006	0.009 ± 0.007	0.003 ± 0.003	0.004 ± 0.006	0.002 ± 0.003	0.026 ± 0.013	0.001 ± 0.002	0.001 ± 0.002	0.039 ± 0.018	0.843 ± 0.321	0.010 ± 0.003	11.992 ± 7.344
**55_DAG42H**	51.955 ± 73.475	5.108 ± 1.652	1.846 ± 0.684	0.049 ± 0.024	0.041 ± 0.021	0.027 ± 0.005	0.020 ± 0.005	0.048 ± 0.023	ND	0.179 ± 0.020	ND	ND	1.326 ± 0.270	0.317 ± 0.170	0.034 ± 0.015	60.950 ± 72.686
**55_DAG63H**	38.507 ± 54.457	8.484 ± 0.480	6.373 ± 1.213	0.033 ± 0.025	0.023 ± 0.017	0.049 ± 0.005	0.025 ± 0.017	0.044 ± 0.004	ND	0.315 ± 0.226	ND	ND	1.910 ± 0.131	0.660 ± 0.025	0.040 ± 0.002	56.462 ± 55.729
**55_DAG7L**	152.712 ± 27.796	13.733 ± 2.777	8.223 ± 1.039	0.348 ± 0.060	0.345 ± 0.067	ND	0.129 ± 0.030	0.060 ± 0.010	0.079 ± 0.008	0.938 ± 0.136	0.087 ± 0.015	ND	1.399 ± 0.163	1.321 ± 0.176	0.050 ± 0.007	179.422 ± 29.477
**55_DAG14L**	8.539 ± 0	0.676 ± 0	0.032 ± 0.024	0.006 ± 0	0.005 ± 0	0.012 ± 0	0.004 ± 0	0.002 ± 0	0.005 ± 0	0.094 ± 0	0.004 ± 0	ND	0.147 ± 0	0.087 ± 0.117	0.044 ± 0.031	9.656 ± 0.172
**55_DAG21L**	4.022 ± 2.192	0.171 ± 0.109	0.025 ± 0.020	ND	ND	ND	ND	ND	ND	ND	ND	ND	0.021 ± 0.008	0.065 ± 0.008	0.370 ± 0.507	4.673 ± 1.775
**55_DAG42L**	ND	3.922 ± 0.026	0.708 ± 0.304	0.038 ± 0.002	0.034 ± 0.001	0.027 ± 0	0.027 ± 0.005	ND	ND	0.217 ± 0.034	ND	ND	0.773 ± 0.512	0.152 ± 0.001	0.279 ± 0.163	6.177 ± 0.385
**55_DAG63L**	ND	ND	0.497 ± 0.260	ND	ND	ND	ND	ND	ND	ND	ND	ND	ND	0.510 ± 0.187	0.252 ± 0.177	1.259 ± 0.594
**SQY_DAG7H**	106.351 ± 36.236	10.762 ± 1.749	8.040 ± 1.950	1.743 ± 0.027	1.631 ± 0.111	0.858 ± 0	0.114 ± 0.016	0.038 ± 0.005	0.493 ± 0.031	1.192 ± 0.481	0.046 ± 0.009	ND	1.200 ± 0.328	3.401 ± 0.155	0.063 ± 0.002	135.933 ± 38.447
**SQY_DAG14H**	46.997 ± 13.598	2.836 ± 1.596	2.925 ± 1.354	0.317 ± 0.125	0.308 ± 0.136	0.061 ± 0.012	0.010 ± 0.014	0.011 ± 0.016	0.041 ± 0.058	0.275 ± 0.114	0.254 ± 0.185	ND	ND	1.647 ± 0.636	0.602 ± 0.257	56.285 ± 16.833
**SQY_DAG21H**	30.053 ± 35.567	4.757 ± 2.452	3.710 ± 2.338	0.241 ± 0.139	0.230 ± 0.141	0.108 ± 0.064	0.033 ± 0.017	0.038 ± 0.020	0.056 ± 0.034	0.360 ± 0.242	0.011 ± 0.006	ND	0.037 ± 0.042	1.910 ± 1.310	0.020 ± 0.011	41.565 ± 38.678
**SQY_DAG42H**	181.903 ± 14.889	13.182 ± 3.421	29.413 ± 8.742	ND	0.136 ± 0.024	0.093 ± 0.022	0.070 ± 0.051	0.115 ± 0.017	0.039 ± 0.012	2.076 ± 0.516	0.085 ± 0.002	0.020 ± 0.028	2.543 ± 0.102	1.032 ± 0.167	1.835 ± 0.558	232.543 ± 24.376
**SQY_DAG63H**	62.419 ± 27.285	7.359 ± 1.398	18.594 ± 9.997	0.071 ± 0.006	0.068 ± 0.011	0.059 ± 0.025	0.087 ± 0.011	0.033 ± 0.023	ND	1.439 ± 0.753	0.046 ± 0.004	ND	1.491 ± 0.494	0.744 ± 0.178	0.037 ± 0.003	92.448 ± 35.193
**SQY_DAG7L**	72.887 ± 11.089	6.271 ± 1.555	3.316 ± 2.154	1.377 ± 1.005	1.176 ± 0.337	0.805 ± 0.199	0.097 ± 0.033	0.036 ± 0	0.405 ± 0.094	0.479 ± 0.172	0.030 ± 0.013	ND	0.332 ± 0.302	1.201 ± 1.409	0.123 ± 0.117	88.534 ± 13.238
**SQY_DAG14L**	28.574 ± 10.788	0.470 ± 0.451	1.028 ± 0.491	0.156 ± 0.029	0.154 ± 0.023	0.051 ± 0.002	ND	ND	ND	0.142 ± 0.035	ND	ND	ND	0.472 ± 0.248	0.786 ± 0.002	31.832 ± 12.683
**SQY_DAG21L**	1.659 ± 0	3.537 ± 3.177	1.783 ± 1.024	0.193 ± 0.112	0.231 ± 0.064	0.074 ± 0.040	0.036 ± 0.020	0.057 ± 0.033	0.047 ± 0.006	0.086 ± 0.050	0.024 ± 0.016	ND	0.171 ± 0.121	0.815 ± 0.449	0.016 ± 0.015	8.730 ± 2.842
**SQY_DAG42L**	72.056 ± 63.528	4.130 ± 3.389	7.688 ± 7.024	0.032 ± 0.023	0.063 ± 0.027	0.062 ± 0.022	0.031 ± 0.004	0.056 ± 0.031	ND	0.592 ± 0.536	0.063 ± 0.027	ND	0.858 ± 1.116	0.602 ± 0.145	0.373 ± 0.488	86.605 ± 73.676
**SQY_DAG63L**	28.764 ± 20.312	3.780 ± 1.187	4.151 ± 0.220	0.060 ± 0.005	0.056 ± 0.003	0.041 ± 0.001	0.058 ± 0.011	ND	ND	0.358 ± 0.013	0.034 ± 0.005	ND	0.296 ± 0.377	1.074 ± 0.101	0.301 ± 0.389	38.973 ± 18.208

DAG, day after germination; ‘H’ represents taproot tissue; ‘L’ represents leaf tissue; ‘ND’, not detected by LC-MS/MS. The data were expressed as the mean ± standard deviation (SD) (n = 3).

To provide information for developing functional foods of radish in the near future, the dynamic accumulation patterns of GSLs in ‘SQY’ and ‘55’ were investigated. In the ‘SQY’, the content of total GSLs in both leaf and taproot tissues exhibited a similar fluctuation pattern, namely, falling at 14 DAG and 21 DAG, then increasing at 42 DAG, and decreasing again at 63 DAG (**Figure S3**). These findings suggested that before the primary thickening stage (14 DAG to 42 DAG), the GSLs might be mainly derived from radish seed, and the biosynthesis ability of GSLs reached the maximum at the secondary thickening stage (42 DAG). However, two different accumulation patterns of total GSLs were found in the leaves and taproots of the ‘55’. The accumulation patterns of GSLs in taproots of ‘SQY’ and ‘55’ showed a similar tendency, while the content of total GSLs displayed a continuous decrease in leaves of ‘55’ during the five developmental stages. Moreover, we further analyzed the accumulation pattern of four major GSLs of GRH, GER, GRE, and 4MGBS (**Figure S4**). During the whole vegetative growth stages, three aliphatic GSLs including GRH, GER, and GRE in leaves and taproots exhibited a fluctuation pattern, especially GRH. However, 4MGBS, indolic GSL, showed different accumulation patterns between leaves and taproots (**Figure S4D**).

### 3.4 Proposed biosynthetic pathways and secondary modifications of GSLs in radish

**Figure 5:**
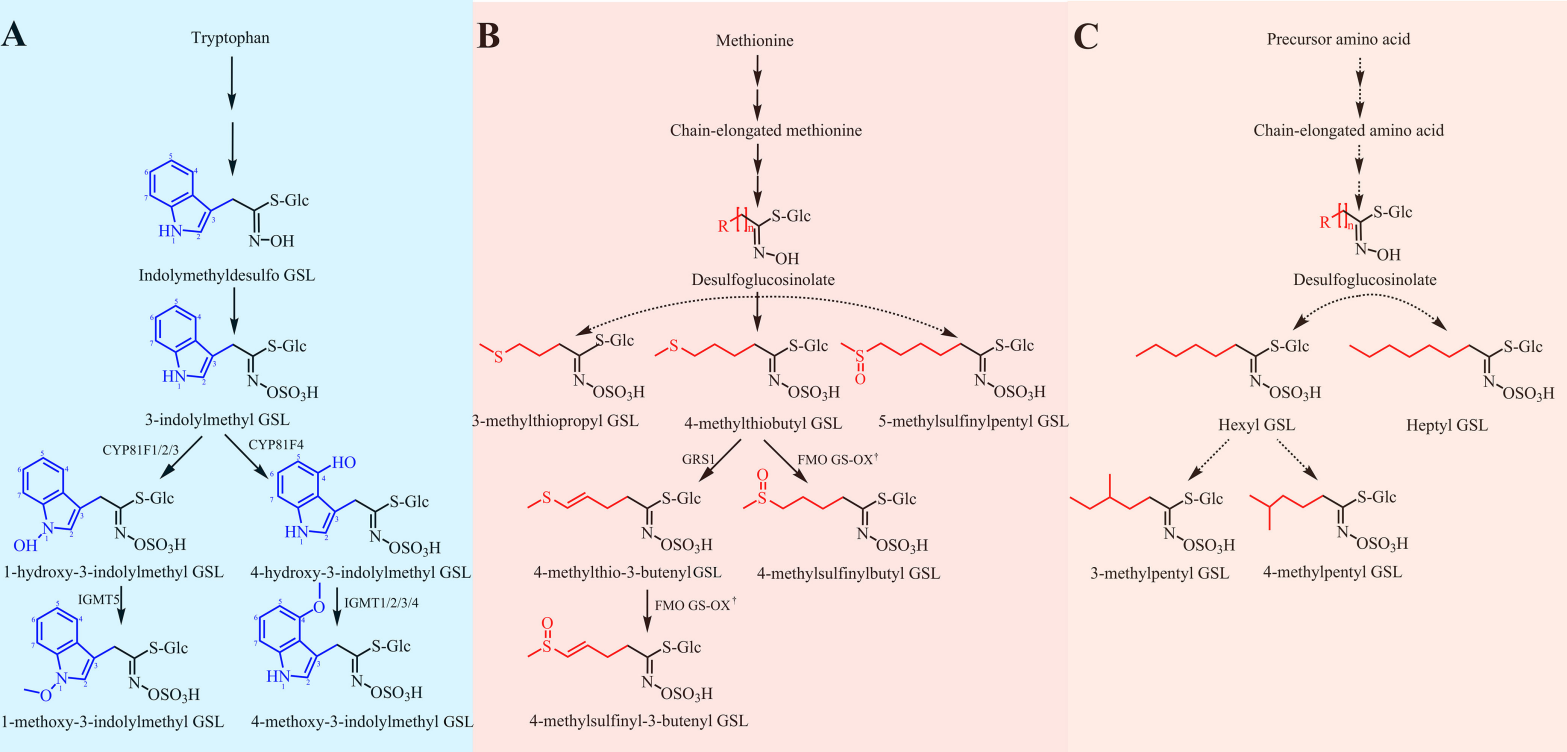
Proposed biosynthetic pathways of GSLs in radish. **(A)** The biosynthetic pathway of indolic GSLs. The biosynthetic pathway was proposed referring to the previous report (Wang et al., 2022). **(B)** The biosynthetic pathway of methionine-derived GSLs. The biosynthetic pathway was referred to two previous investigations (Kakizaki et al., 2017; Li et al., 2022). **(C)** An unknown biosynthetic pathway of aliphatic GSLs. Dotted arrows represent some unknown steps. † represents some one homologous of FMO GS-OXs. GRH, 4-methylthio-3-butenyl GSL; GRE, 4-methylsulfinyl-3-butenyl GSL; GER, 4-methylthiobutyl GSL; GRA, 4-methylsulfinylbutyl GSL; GIV, 3-methylthiopropyl GSL; GAL, 5-methylsulfinylpentyl GSL; GBS, 3-indolylmethyl GSL; 4HGBS, 4-hydroxy-3-indolylmethyl GSL; 1HGBS, 1-hydroxy-3-indolylmethyl GSL; 4MGBS, 4-methoxy-3-indolylmethyl GSL; NEO, 1-methoxy-3-indolylmethyl GSL.

For breeding purposes, we mainly examined the biosynthesis of aliphatic and indolic GSLs. Based on our findings of intact GSL structures and previous relevant reports, we proposed three biosynthetic pathways of GSLs in radish (**Figure 5**). The major difference in radish-specific GSL biosynthesis was in the final step, a secondary modification. In radish, four reported secondary modifications of GSLs, including oxygenations, alkenylations, hydroxylations, and methoxylations, were discovered, two of which, oxygenations and alkenylations, were observed in aliphatic GSLs (**Figure 5C**). Based on different substrates, GRH and GER were respectively oxidized by flavin monooxygenases glucosinolate S-oxygenase (FMO GS-OX) into sulfinyl GSLs of GRE and GRA. For five indolic GSLs, we also identified two secondary modifications hydroxylation and methoxylation (**Figure 5C**). GBS, as upstream indolic GSL, was hydroxylated firstly by CYP81F members to produce 1-hydroxyglucobrassicin (1HGBS) and 4HGBS. The position of hydroxyl groups, 1-OH and 4-OH, was controlled by CYP81F members in Arabidopsis (Pfalz et al., 2009; Pfalz et al., 2011). Then, the indolic GSLs, 1HGBS and 4HGBS were respectively methylated by methyltransferases (IGMT1 and IGMT2) to synthesize NEO and 4MGBS, which were known as methoxylations. Taken together, our proposed GSL biosynthesis pathways in radish will lay a foundation for revealing metabolic mechanisms and molecular design breeding in the future.

## 4 Discussion

Radish is an outstanding plant resource that effectively serves the purpose of both a medicinal agent and a nutritional source simultaneously.Numerous naturally occurring bioactive compounds in radish, such as GSLs and their degradation products, have sparked intense interest among academics (Gamba et al., 2021). In the previous study, high-performance liquid chromatography (HPLC) was generally employed to detect GSLs in radish which required desulfonation to decrease the strong polarity of the thioglucosyl group (-SGlc). The process of desulfonation frequently results in impaired structures, insufficient reactions, diminished concentrations of GSL, and significant time expenditure. (Glauser et al., 2012). Although various desulfur-GSL structures have been characterized in radish (Ishida et al., 2015; Yi et al., 2016; Wang et al., 2022), accurate qualitative analysis of intact GSL profiles has rarely been reported. Therefore, a reliable and accurate approach to profile intact GSLs in radish is urgently needed. Considering this, we employed a novel LC-MS/MS method to promote the traditional HPLC method for establishing more complete GSL profiles in radish.

The GSLs exhibited similar cleavage patterns under their regular molecular structure of GSLs. Several common fragments were produced under the HCD model, among which four of them showed a high response. Furthermore, since each GSL component has a unique side chain, each substance displayed varied polarity and produced distinct side chain fragments. These results will provide available information to characterize intact GSLs in radish. With the aid of our integrated LC-MS/MS method, a total of 15 intact GSLs in radish were identified, including 10 aliphatic GSLs and five indolic GSLs. There is no doubt that the radish-specific GRH, which accounts for 67.55%-85.24% of the total GSLs, is the predominant GSL component in radish taproots (**Table S3**).

Generally, the biosynthesis of GSLs involves three steps: (1) side chain elongations (only for methionine), (2) formation of the core structures, and (3) secondary modifications of the side chain (Augustine and Bisht, 2017). Based on previous studies, the major difference in radish-specific GSL biosynthesis occurred in the last step. Different from Arabidopsis, GER in radish was primarily dehydrogenated by *GRS1*(*Glucoraphasatin Synthase 1*), which encodes a Fe (II)-dependent dioxygenase, to generate unsaturated GRH (Kakizaki et al., 2017). Besides, although 3-butenyl GSL (Gluconapin, GNP) and PRO were previously reported in Brassica plants, we were unable to discover these two typical aliphatic GSLs in our investigation. Interestingly, the absence of RsAOP2 and RsAOP3 dioxygenases, which are linked to GNP formation, has been found in two radish reference genomes (Mitsui et al., 2015; Li et al., 2022). Fortunately, these variations reduced the contents of PRO and its animal-harming degradation product oxazolidine-2-thiones (Petersen et al., 2018). Additionally, a small amount of aromatic GSL gluconasturtiin has been reported to be discovered in the wild radish and some Korean radish cultivars (Malik et al., 2010; Jeon et al., 2022). However, no aromatic GSLs were detected in this study. This unexpected occurrence suggested that GSL profiles varied among different radish germplasms. These findings indicated the radish-specific GSL biosynthetic pathway differed from that of GSL biosynthesis in Arabidopsis and other Brassicaceae plants.

GSLs and their degradation products were reported to be responsible for the spicy taste of radish as well as for health promotion, particularly in terms of their anticancer effects. Based on the accumulation patterns of GSLs during the vegetative growth period, two strategies for generating GSL-rich foods and extraction of GSLs were proposed for the further utilization of the bioactive substances of GSLs in radishes. One sprout-based strategy is involves cultivating GSL-rich radish sprouts for consumption. Currently, GSL-rich vegetables have been widely consumed worldwide (Aloo et al., 2021). For instance, broccoli sprouts have been reported to improve gut health and repress tumor necrosis factor α *in vivo* cell model (Ferruzza et al., 2016). The taproot-based strategy is another tactic. According to the accumulation pattern of GSLs in radish, the 42 DAG and its proximate period should be a suitable harvest time for GSL-rich vegetable and/or GSL extraction. Our findings will furnish valuable insights for enhancing the productivity of individual GSL in radish and developing utilization of natural bioactive components.

## 5 Conclusion

In this study, we performed qualitative and quantitative analyses of the intact GSLs in radish, including the chemical structural characteristics, fragmentation patterns, dynamic contents, and proposed biosynthetic pathway of GSLs using the high-quality mass spectrometry data. A total of 15 intact GSLs were identified from two radish accessions ‘SQY’ and ‘55’. At the 42 DAG stage, the ‘SQY’ taproot had the highest GSL content, with GRH making up the majority of those over 67.55% of the total GSLs. The various GSL accumulation patterns across taproot and leaf tissues of ‘SQY’ and ‘55’ during five vegetative growth stages were discovered using a comparative metabolic analysis of GSLs. Based on these findings, sprout-based and taproot-based strategies were proposed to develop GSLs-rich functional foods. In addition, the GSL biosynthetic pathway in radish was depicted by combining our findings with existing research. Our study provides an integrated method of UHPLC-HRMS/MS in combination with UHPLC-QqQ-MS/MS for the identification and quantification of intact GSLs in radish, as well as lays the theoretical foundation for further development of GSL-rich functional foods from radish in near future.

## Data availability statement

The original contributions presented in the study are included in the article/**Supplementary Material**. Further inquiries can be directed to the corresponding authors.

## Author contributions

CY: Conceptualization, Data analysis, Writing–original draft. YH: Investigation, Data analysis. SZ: Plant management, Review & editing. LC: Resources. ZJ: Resources. ZP: Resources. XL: Resources, Review & editing. YL: Conceptualization, Data analysis, Writing-review & editing. ZQ: Supervision, Funding acquisition, Project administration, Writing-review & editing. All authors contributed to the article and approved the submitted version.
