# Supplementary material for: Dynamic profiling of intact glucosinolates in radish by combining UHPLC-HRMS/MS and UHPLC-QqQ-MS/MS
Source: Front Plant Sci. 2023 Jul 5;14:1216682. doi: 10.3389/fpls.2023.1216682 (PMC10354559; doi:10.3389/fpls.2023.1216682)
Supplement: Supplementary file 1 [file DataSheet_1.docx]

Supplementary Material

Dynamic profiling of intact glucosinolates in radish by combining UHPLC-HRMS/MS and UHPLC-QqQ-MS/MS

**
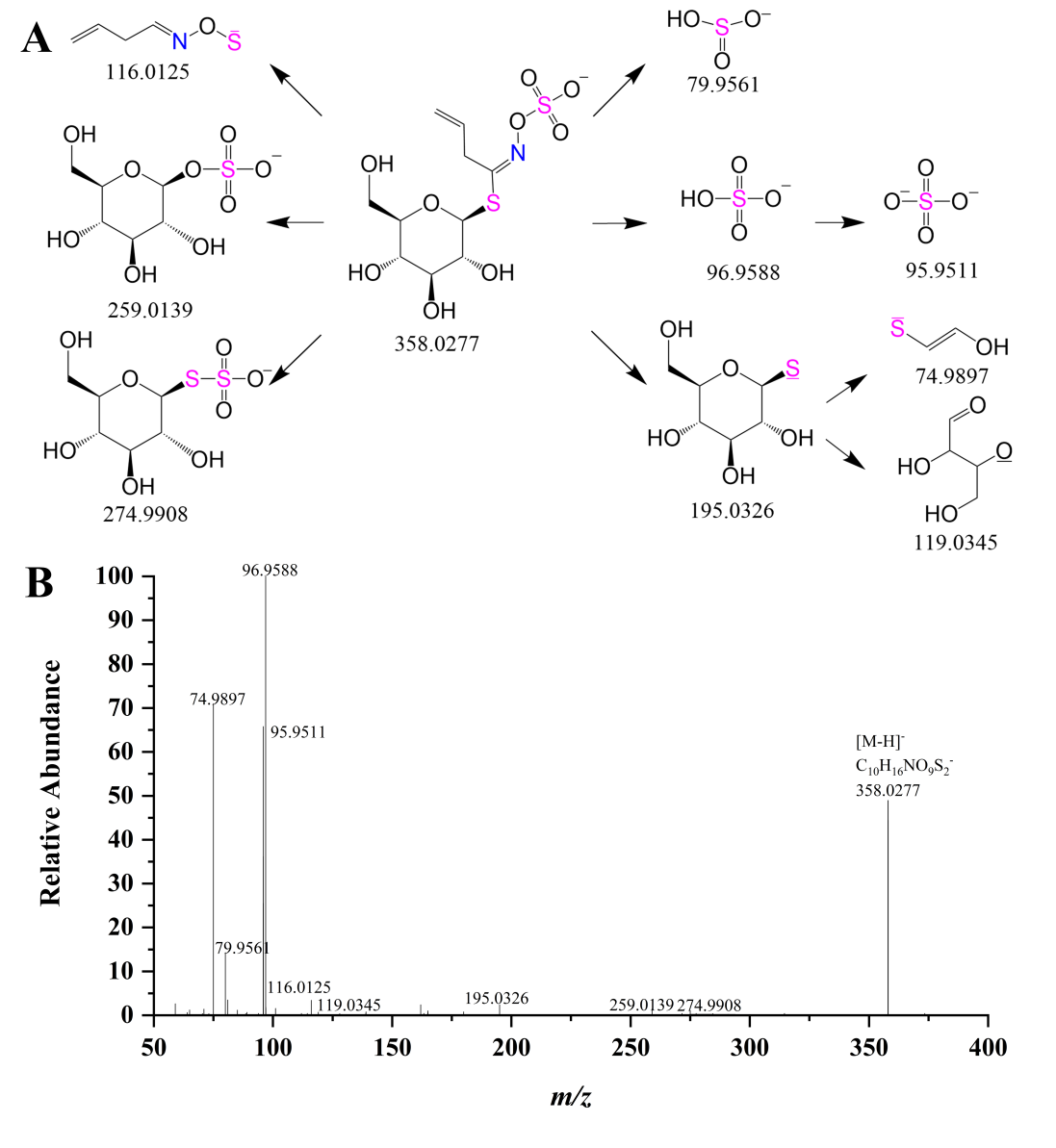
**

**Fig. S1. Cracking products (A) and MS/MS spectrum (B) of sinigrin.**

**
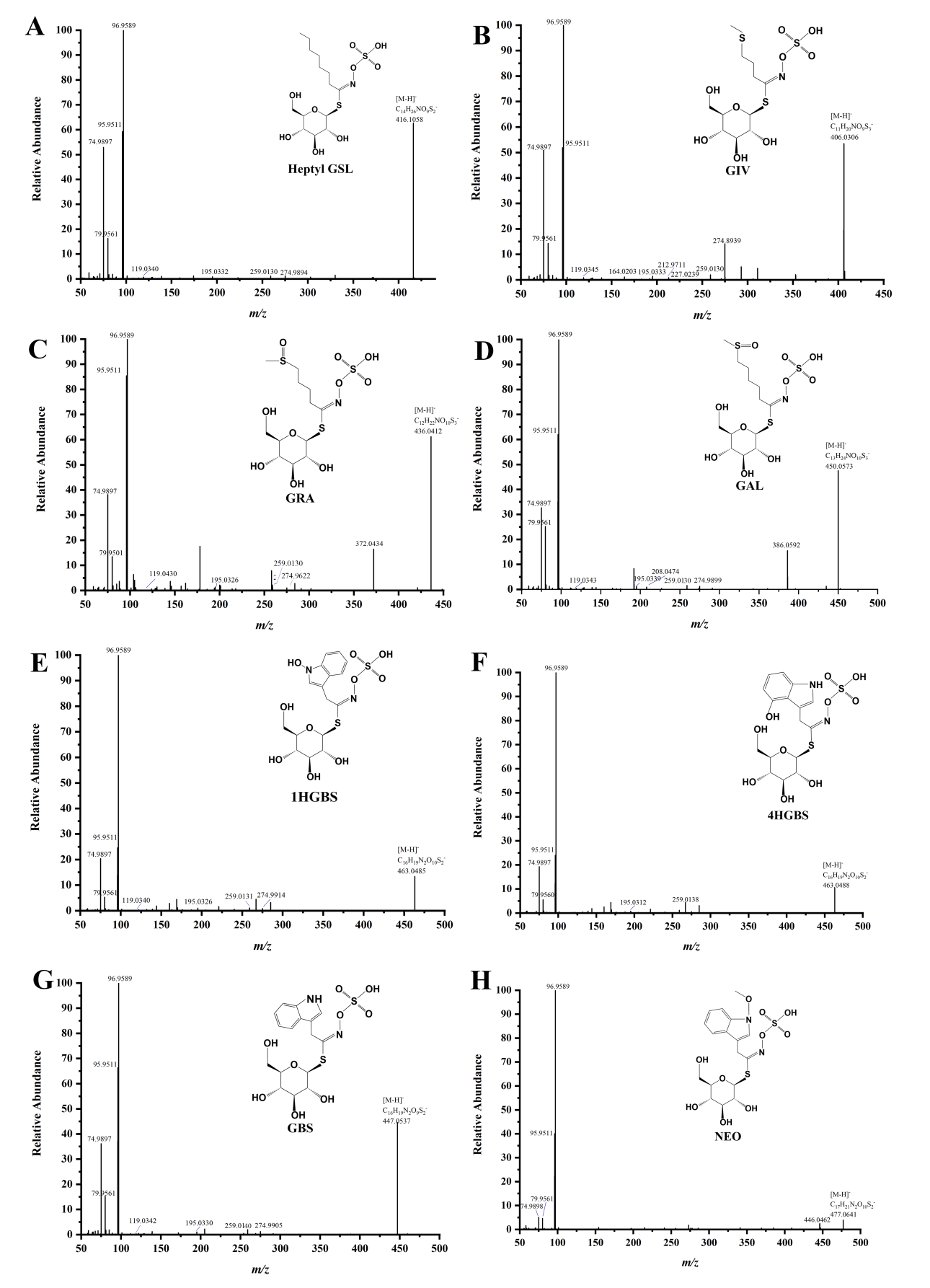
**

**Fig. S2. MS/MS spectrum of Heptyl GSL (A), GIV (B), GRA (C), GAL (D), 1HGBS (E), 4HGBS (F), GBS (G), and NEO (H).**


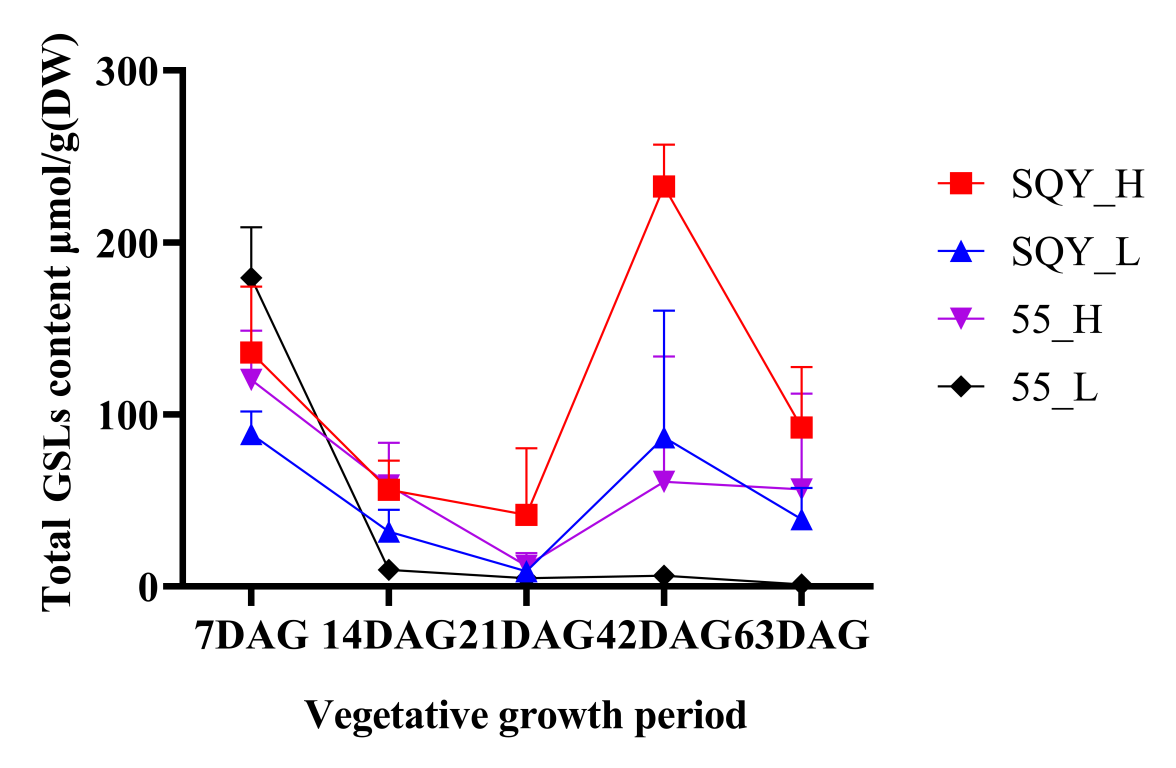


**Fig. S3. Accumulation pattern of the content of total GSLs in ‘SQY’ and ‘55’.** ‘H’, taproot tissue; ‘L’, leaf tissue; DAG, the day after germination; The numbers represent the day of collected samples.


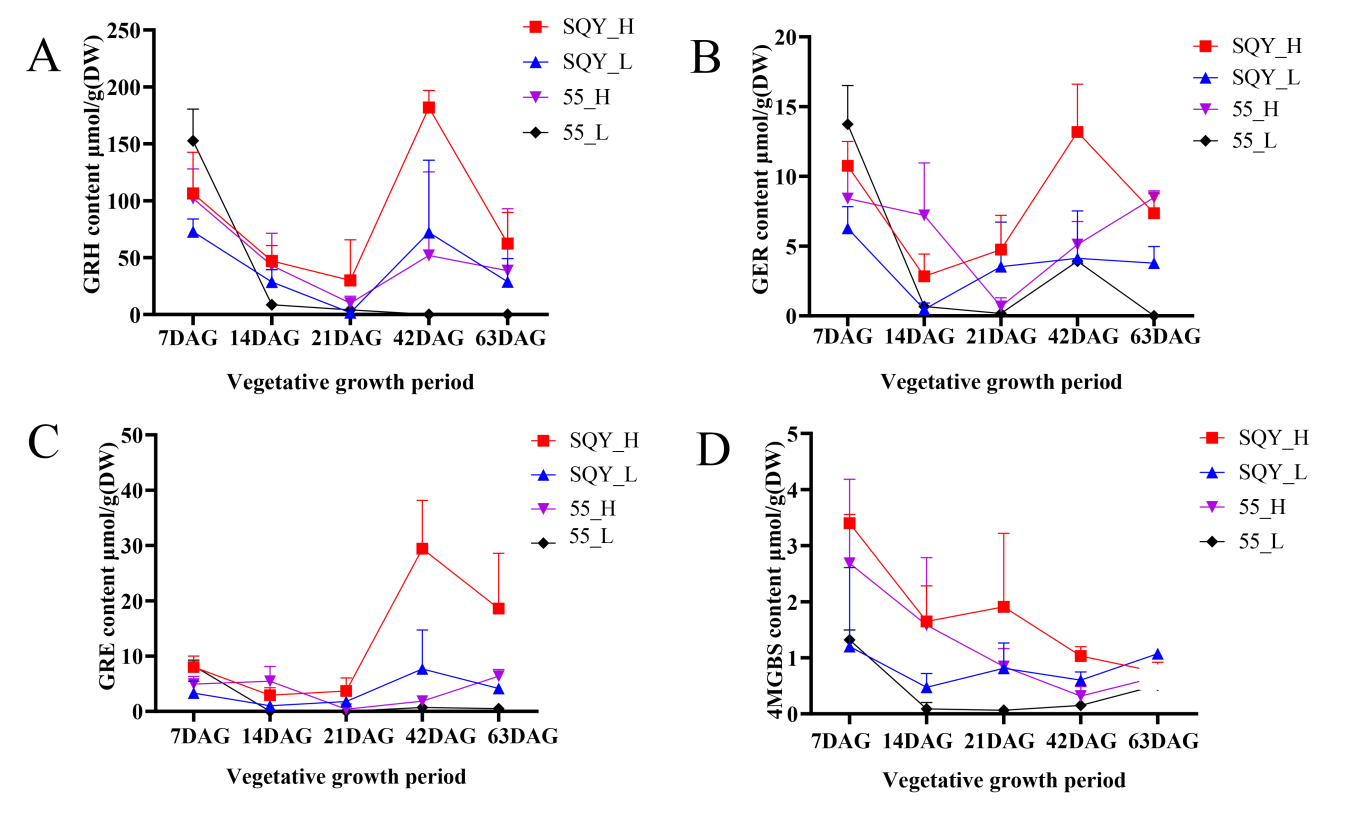


**Fig. S4.** **The variation pattern of the contents of GRH (A), GER (B), GRE (C), and 4MGBS (D) in two radish accessions during the vegetative growth period.** ‘H’ represents taproot tissue; ‘L’ represents leaf tissue; DAG represents the day after germination; the numbers represent the day of collected samples.

**Table S1 A complete list of selective reaction monitoring compound-relative parameters.**

| **Compound Name** | **RT/min** | **Precursor (m/z)** | **RT Window /min** | **Polarity** | **Product (m/z)** | **Collision Energy/eV** |
| --- | --- | --- | --- | --- | --- | --- |
| SIN | 1.95 | 358.027 | 1 | Negative | 96.959 | 23 |
| GRA | 2.10 | 436.041 | 1 | Negative | 96.959 | 23 |
| GRE | 2.25 | 434.025 | 1 | Negative | 96.959 | 23 |
| GAL | 3.93 | 450.057 | 1 | Negative | 96.959 | 23 |
| 1HGBS | 4.62 | 463.049 | 1 | Negative | 96.959 | 23 |
| GIV | 4.76 | 406.031 | 1 | Negative | 96.959 | 23 |
| 4HGBS | 5.63 | 463.049 | 1 | Negative | 96.959 | 23 |
| GER | 5.66 | 420.046 | 1 | Negative | 96.959 | 23 |
| GRH | 5.73 | 418.030 | 1 | Negative | 96.959 | 23 |
| GBS | 6.00 | 447.054 | 1 | Negative | 96.959 | 23 |
| 4MGBS | 7.20 | 477.064 | 1 | Negative | 96.959 | 23 |
| 3-methylpentyl GSL | 7.85 | 402.090 | 1 | Negative | 96.959 | 23 |
| 4-methylpentyl GSL | 8.09 | 402.090 | 1 | Negative | 96.959 | 23 |
| NEO | 8.10 | 477.064 | 1 | Negative | 96.959 | 23 |
| Hexyl GSL | 8.82 | 402.086 | 1 | Negative | 96.959 | 23 |
| Heptyl GSL | 9.37 | 416.106 | 1 | Negative | 96.959 | 23 |

Table S2 Comparison of time consumption between desulfonation method and LC-MS/MS method

| Method | Extraction | Preparation of Sulfatase and DEAE column | Desulfonation process | Detection time | Total time consumption |
| --- | --- | --- | --- | --- | --- |
| Desulfonation (HPLC) method | ≈30 min | Need, >12 hours | Need, >16 hours | >35 min | > 29 hours |
| LC-MS/MS method | ≈60 min | No need | No need | 14 min | ≈ 1.5 hours |

Note: the time consumption of desulfonation method refers to the previous studies (Kim et al., 2013; Yi et al. at 2016).s

**Table S3 The proportion of GRH, GRE, GER, and 4MGBS in total GSLs.**

|  | GRH in total GSL (%) | GRE in total GSL (%) | GER in total GSL (%) | 4MGBS in total GSL (%) |
| --- | --- | --- | --- | --- |
| SQY_D7H | 78.26 | 5.92 | 7.92 | 2.50 |
| SQY_D14H | 83.88 | 5.22 | 5.06 | 2.93 |
| SQY_D21H | 72.32 | 8.93 | 11.45 | 4.60 |
| SQY_D42H | 78.25 | 12.65 | 5.67 | 0.44 |
| SQY_D63H | 67.55 | 20.12 | 7.96 | 0.80 |
| SQY_D7L | 82.35 | 3.75 | 7.09 | 1.36 |
| SQY_D14L | 89.77 | 3.23 | 1.48 | 1.48 |
| SQY_D21L | 19.06 | 20.48 | 40.63 | 9.33 |
| SQY_D42L | 83.26 | 8.88 | 4.77 | 0.69 |
| SQY_D63L | 73.87 | 10.66 | 9.71 | 2.76 |
| 55_D7H | 84.96 | 4.13 | 7.00 | 2.24 |
| 55_D14H | 73.35 | 9.31 | 12.26 | 2.69 |
| 55_D21H | 83.02 | 3.36 | 5.66 | 7.03 |
| 55_D42H | 85.24 | 3.03 | 8.38 | 0.52 |
| 55_D63H | 68.20 | 11.29 | 15.03 | 1.17 |
| 55_D7L | 85.15 | 4.58 | 7.66 | 0.74 |
| 55_D14L | 88.47 | 0.33 | 7.00 | 0.90 |
| 55_D21L | 86.05 | 0.53 | 3.66 | 1.39 |
| 55_D42L | 0 | 11.46 | 63.49 | 2.46 |
| 55_D63L | 0 | 39.46 | 0 | 40.50 |
